# Analysis of Emergency Department Presentations due to Injuries From Motor Vehicle Crashes and Pedestrian Strikes

**DOI:** 10.7759/cureus.9468

**Published:** 2020-07-30

**Authors:** Canan Akman, Tolgahan Kuru

**Affiliations:** 1 Emergency Medicine, Canakkale Onsekiz Mart University Faculty of Medicine, Canakkale, TUR; 2 Orthopaedics and Traumatology, Canakkale Onsekiz Mart University, Canakkale, TUR

**Keywords:** emergency department, motor vehicle accident, pedestrian struck, holiday, alcohol

## Abstract

Objective

The objective of this study was to analyze the causes and outcomes of presentations to the emergency department (ED) due to injuries from motor vehicle crashes and pedestrian strikes along several parameters.

Methods

Data from 798 patients who were injured due to motor vehicle crashes or pedestrian strikes that occurred in Canakkale Province were retrospectively analyzed. Patient demographic data such as age and gender, emergency service outcomes, and the consulted clinics were also recorded. Distributions of the crashes by weekdays or weekends, national and religious holidays, official holidays, and Ramadan month were comparatively analyzed.

Results

A total of 253 people injured in motor vehicle crashes and 395 people injured in pedestrian strikes were directly brought to the ED from the crash or strike scene. While 656 patients were discharged from the ED, 142 patients were referred to other clinics for surgery. A total of 538 crashes occurred on weekdays and 206 on weekends, and 54 crashes occurred during official and religious holidays. Most crashes occurred in summer, and the second most occurred in autumn. The rate of pedestrian strikes that occurred in summer and autumn was statistically significantly higher than the rate of injuries from motor vehicle crashes observed in the same seasons. The majority of the weekend crashes were caused by persons who had not consumed alcohol.

Conclusion

Traffic crashes occur more commonly among young men and in the summer season, while national, official, and religious holidays do not seem to play a role in the frequency of traffic crashes.

## Introduction

Intense emergency department (ED) use is a global public health problem affecting access to care and quality of care [1]. Evidence indicates that the use of emergency healthcare services is increasing due to injuries from motor vehicle crashes. However, the capacity of EDs has increased to meet this high demand because it is difficult to establish a balance between ED capacity and necessity [2]. Overly crowded EDs lead to undesired outcomes for patients, providers, healthcare systems, and society. Crowds in EDs at or near capacity can cause delayed care and increased waiting times, increased workload of emergency specialists, and increased morbidity and mortality [3]. Therefore, a sustainable analysis of ED presentations and the reasons for ED visits will contribute to the development of new strategies for decreasing the intensity of ED use.

A traffic crash is defined as an event resulting in death, injury, or financial loss due to a collision of one or more vehicles or a vehicle and pedestrian (i.e., a pedestrian strike [PS]). Traffic crashes can account for a substantial portion of ED visits, which is a difficult problem to overcome in our country and worldwide. According to the 2018 World Health Organization (WHO) data, more than 55 million people get injured each year from motor vehicle crashes and 1.35 million people die from motor vehicle-related injuries [4]. By 2030, traffic crashes may be the fifth leading cause of death worldwide, with a projected 2.4 million people dying from crash-related injuries [5]. Understanding the reasons for traffic crashes and various factors related to patients and injuries will be helpful both for the determination of measures to be taken before and after these crashes and for a healthier analysis of presentations to EDs due to crashes.

Traffic crashes occurring on roads involve pedestrians and/or vehicles. Vehicle crashes are defined as motor vehicle injuries (MVIs) and collision with pedestrians as PS [6]. Etiological analysis of both MVIs and PSs and developing solutions based on these analyses will play an important role in reducing the intensity of ED use.

Thus far, factors affecting ED visits have been investigated in numerous studies [7-9]. However, given the dynamic nature of traffic crashes, changing parameters, and capacity problems of EDs, studies performing analysis of the etiological analysis of traffic crashes from the view of EDs will be continuously needed. Therefore, the objective of this study was to analyze the causes and outcomes of the presentation to the ED due to MVIs and PSs in terms of several parameters.

## Materials and methods

Data of 798 patients aged between 18 and 81 years who were injured due to motor vehicle crashes or PS that occurred in Canakkale Province or out of the provincial borders and presented to the ED of our hospital between December 2019 and May 2020 were obtained from the hospital registry system and retrospectively analyzed. Patients who were injured due to traffic crashes in Çanakkale city center or out of the provincial borders, referred from an outer center, or directly transferred to our hospital from the crash or PS scene were included in the study. Patients aged younger than 18 years, those with missing data for use in the study, foreign national patients, and those who left the ED on their own request before receiving a formal discharge or referral were excluded from the study. Also, patients who were dead on arrival to the ED were excluded.

Patients’ demographic data such as age and gender, emergency service outcome (discharge, referral for surgery), and the consulting clinics were also recorded.

MVIs were defined as injuries from crashes caused by a vehicle to its own occupants or those of another vehicle, while collisions of a vehicle with a person not occupying another vehicle were defined as PSs. In addition, distributions of the crashes by weekdays or weekends, national and religious holidays, official holidays, and Ramadan month were comparatively analyzed (Table 1). 

**Table 1:** National, religious, and official holidays

Distribution of Crashes by Days and Holidays	
National Holidays	
April 23	National Sovereignty and Children's Day
May 1	Workers' Day
May 19	The Commemoration of Ataturk, Youth, and Sports Day
July 15	Democracy Holiday
August 30	Victory Day
October 29	Republic Day
Religious Holidays	
	Ramadan Feast
	Feast of Sacrifice
Official Holidays	
	New Year's Day

Ramadan is a holy month in Islam. Muslims fast during Ramadan month. Some drivers become more nervous and stressed due to fasting. Therefore, in addition to holidays, we analyzed injuries due to traffic crashes during Ramadan and investigated whether the number of crashes increased during this period.

Distribution of MVIs and PSs according to seasons and months was examined. Alcohol intake status was investigated in traffic crashes and compared between the groups. Also, the form of arrival to the ED was examined in two groups as the injured people who came with a referral from another center and those brought directly from the crash or strike scene. The occurrence of the crashes in Çanakkale city center or out of the provincial borders was analyzed.

Patients included in the study were first appropriately stabilized during the first admission, triage was performed, and the necessary emergency treatments were provided according to the general status of the patients. A detailed history was received from the patients and/or their relatives, including age, gender, type of the accident (MVI or PS), mechanism of the accident, whether the seat belt was fastened, and alcohol intake status. Associated visceral injury, the type of orthopedic injury, and affected body regions were also recorded.

Ethics considerations

Before the beginning of the study, the necessary approval was received from the local ethics committee of our hospital. The study was conducted in accordance with the ethical principles of the Declaration of Helsinki. Because the study was retrospective, informed consent forms were waived.

Statistical analysis

Data obtained in the study were statistically analyzed using IBM SPSS Statistics for Windows, Version 22.0. (Armonk, NY: IBM Corp.). The normality of the data was tested with the Kolmogorov-Smirnov method. Continuous variables are expressed as mean ± standard deviation, median, minimum, and maximum descriptive statistics. Categorical variables are given as number and percentage. Analysis of the data was performed using Student’s t-test and chi-square test. The relationships between the examined parameters were studied using Spearman’s correlation analysis. A p-value <0.05 was considered statistically significant.

## Results

A total of 798 patients involved in traffic crashes in Çanakkale city center or out of the provincial borders, referred from another center or brought directly from the scene to the ED department of our hospital between December 2019 and May 2020, were included in the study.

Of all patients, 202 (25.3%) were women and 596 (74.7%) were men. The mean age of the patients was 39.9±15.9 years; the median age was 36 years (min-max: 18-81 years). A total of 150 patients (18.8%) were referred from another center, and 648 (81.2%) were brought directly from the crash scene. A total of 323 patients (40.5%) had MVI, while 475 (59.5%) were injured in a PS. Of the MVIs, 70 patients (21.7%) were referred from another center, while 253 (78.3%) were brought directly from the scene. In PS injuries, 80 patients (16.5%) were referred from another center, while 395 (83.2%) were brought directly from the scene. While 656 patients (82.2%) were discharged from the ED, 142 patients (17.8%) were referred to other clinics for surgery. Accordingly, patients who were injured in a traffic crash and referred to other clinics were most commonly referred to the orthopedics (14.8%), neurosurgery (8.1%), and thoracic diseases (2.3%) clinics. The distribution of the clinics where the patients were referred is given in Table 2.

**Table 2:** Distribution of the other clinics where the patients were referred from the emergency department

Clinics		N	%
Consulted Clinics	No Surgical Procedure	511	64.0
	Neurosurgery	65	8.1
	Thoracic Diseases	18	2.3
	General Surgery	8	1.0
	Urology	2	0.3
	Orthopedics	118	14.8
	Plastics	8	1.0
	Ophthalmology	6	0.8
	Cardiovascular Surgery	1	0.1
	Ear, Nose, and Throat	1	0.1
	Obstetrics and Gynecology	2	0.3
	Neurosurgery + Orthopedics	14	1.8
	Neurosurgery + Thoracic Diseases	4	0.5
	Neurosurgery + Thoracic Diseases + Orthopedics	2	0.3
	Thoracic Diseases + Orthopedics	7	0.9
	Plastics + Ophthalmology	1	0.1
	Neurosurgery + Ophthalmology	3	0.4
	Physical Therapy and Rehabilitation + Ophthalmology	1	0.1
	General Surgery + Orthopedics	6	0.8
	Orthopedics + Physical Therapy and Rehabilitation	1	0.1
	Neurosurgery + General surgery + Orthopedics + Obstetrics and gynecology	1	0.1
	Neurosurgery + Orthopedics + Ophthalmology	1	0.1
	Urology + Orthopedics	3	0.4
	Neurosurgery + Plastics	2	0.3
	Neurosurgery + Plastics + Ophthalmology	1	0.1
	Plastics + Ophthalmology	1	0.1
	Neurosurgery + Thoracic Diseases + General Surgery + Orthopedics	1	0.1
	Orthopedics + Plastics + Ophthalmology + Ear, Nose, and Throat	1	0.1
	General surgery + Urology	1	0.1
	Orthopedics + Plastics	1	0.1
	Thoracic Diseases + General Surgery	1	0.1
	Thoracic Diseases + Plastics	1	0.1
	Neurosurgery + Thoracic Diseases + General Surgery	1	0.1
	Thoracic Diseases + Ophthalmology	3	0.4
	Total	798	100.0

When characteristics of the crashes were examined, 419 (52.5%) occurred in the city center and 379 (47.5%) occurred out of the provincial borders. There was no statistically significant difference in the number of crashes that occurred in Çanakkale city center and those observed in the out of the provincial borders for holidays vs. non-holidays (p=0.645).

**Figure 1:**
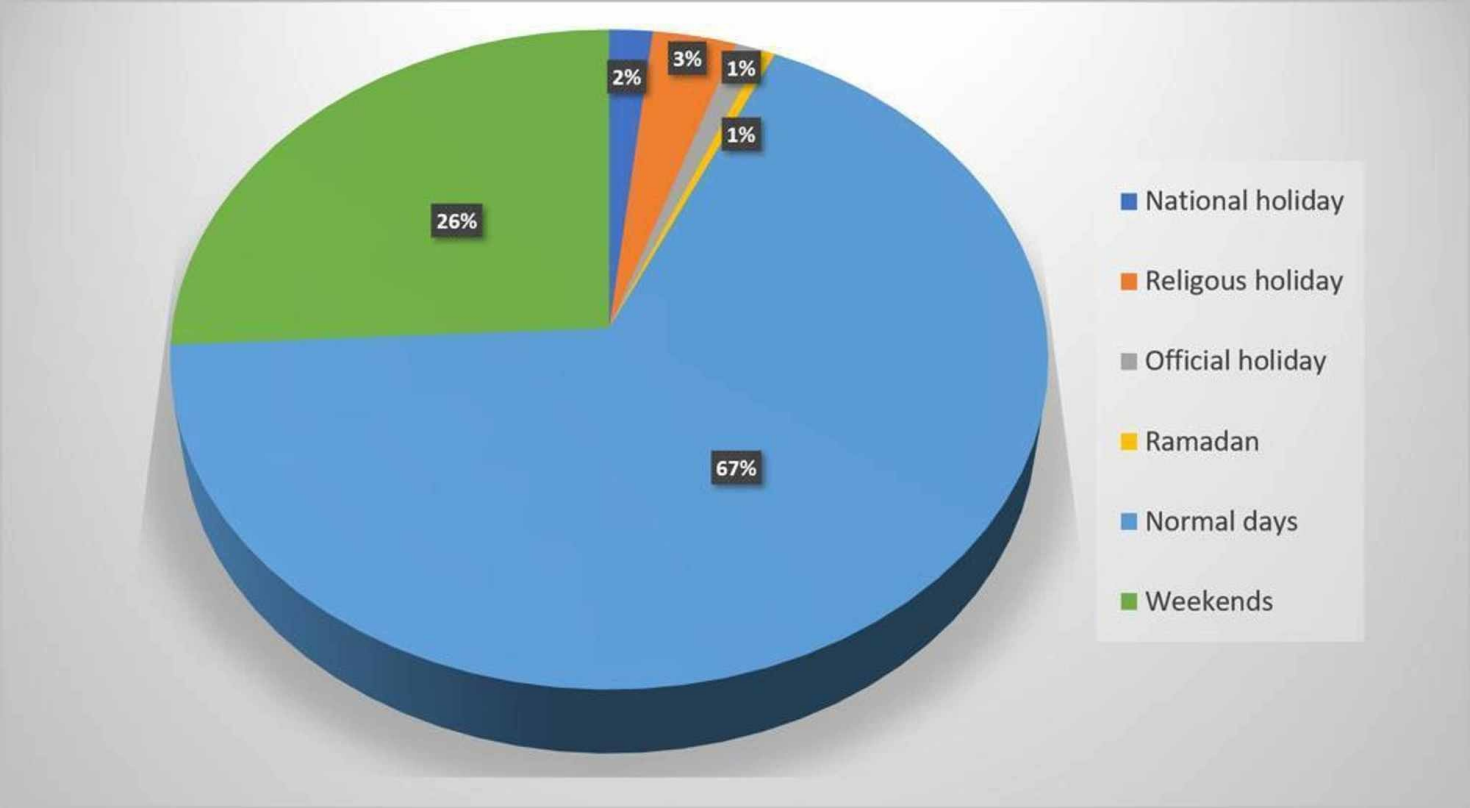
Distribution of traffic accidents according to normal days and holidays

A total of 538 crashes (67.4%) occurred on weekdays and 206 (25.8%) on weekends, and 54 crashes (6.8%) occurred during official and religious holidays (Figure 1).

Of the 260 crashes during holidays (national and religious holidays, weekends), 130 (50%) occurred in the city center and 130 (50%) outside of the province. Also, while the Ramadan month was separately evaluated, only four crashes (0.5%) occurred in this holy month. Traffic crashes with alcohol intake were significantly decreased in the Ramadan month and religious holidays (alcohol (+): 20%; alcohol (-): 80%). No statistically significant difference was found between the patients discharged from the ED and those referred to the other clinics for surgery in terms of the occurrence of the accident in non-holidays vs. holidays (p=0.448).

**Figure 2:**
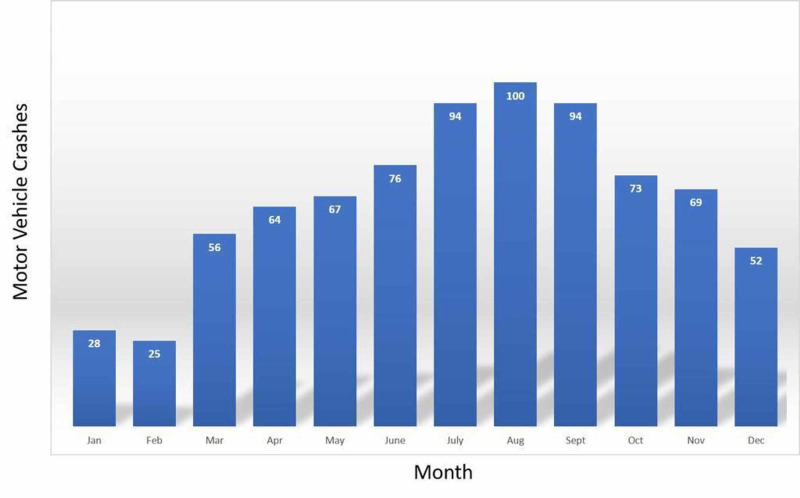
Distribution of traffic crashes by months

When the traffic crashes were evaluated by seasons and months, we noted that 269 (33.7%) crashes occurred in summer, 240 (30.1%) in autumn, 183 (22.6%) in spring, and 106 (13.3%) in winter. There was a significant correlation between the season in which the crashes occurred and the rates of crashes in non-holidays and holidays (r=70.1, p<0.05). Again, a statistically significant correlation was observed between the season in which the crashes occurred and the rates of crashes seen in Çanakkale city center or out of the provincial borders (r=9.1, p=0.028). The crashes that occurred in the city center were most commonly seen in summer and autumn, while the crashes observed out of the provincial borders most commonly occurred in autumn and summer, respectively. There was a statistically significant difference between MVIs and PSs in terms of the season in which the accident occurred. Accordingly, the rate of PSs that occurred in summer and autumn was statistically significantly higher than the rate of MVIs observed in the same seasons (p=0.024). Among the crashes that occurred on weekends, no statistically significant difference was found between the rates of the crashes observed in the city center and out of the provincial borders in terms of the rates of MVIs and PSs (p=0.218). Again, no statistically significant difference was found between the crashes that occurred in the city center and those observed out of the provincial borders in terms of alcohol intake (p=0.087). Traffic crashes were most observed in July, August, and September, and occurred least frequently in February (Figure 2).

In 682 crashes (85.5%), the driver was alcohol-free, while the driver had consumed alcohol in 116 crashes (14.5%). No statistically significant difference was found between MVIs and PSs in terms of alcohol intake (p=0.216). There was a statistically significant difference between alcohol intake and the crashes that occurred on weekends (r=74.6, p<0.05). A majority of the weekend crashes were caused by persons who did not consume alcohol. Of the cases with alcohol (+), 26 (22.4%) were referred from an outer center, while 90 (77.6%) were directly brought from the scene. Of the cases with alcohol (-), 124 (18.2%) were referred from an outer center, while 558 (81.8%) were directly brought from the scene. A total of 94 patients with alcohol (+) were discharged from the ED, while 22 patients (19%) were referred to the other clinics for surgery. A total of 562 patients (82.4%) with alcohol (-) were discharged from the ED and 120 (17.6%) were referred to the other clinics for surgery.

## Discussion

Injuries from motor vehicle crash events constitute a significant proportion of ED presentations and increase the workload of emergency services. Globally, traffic crashes are the leading cause of injury-related deaths [10]. In the present study, we investigated the changes in the rates of MVIs and PSs according to the city center, out of the provincial borders, weekdays, weekends, national, official and religious holidays, seasons, months, and alcohol intake.

The rates of traffic crashes and the severity of the crashes vary between the sexes. According to a report published by the WHO, more than 75% of deaths from traffic crashes occur in men [11]. Li et al. reported that men historically travel for longer distances, which increases the likelihood of a traffic accident [12]. In a study by Ghadipasha et al., 79.2% of the patients injured in a motor vehicle crash were men [13]. Kual et al. reported the ratio of men to women involved in traffic crashes as 3:1 [14]. Consistently with the literature, our study found that 74.7% of the patients were men. According to the WHO, being young and male are considered factors determining the occurrence of traffic crashes [15]. The higher rate of men involved in crashes in our study and the previous studies may be due to cultural factors that indicate car driving is more commonly performed by men, driving is predominantly undertaken by men as a profession, and that men are more active compared to women. In addition, men more commonly tend to engage in risky behaviors such as driving while drinking alcohol, overdriving, and not using seat belts.

In a study by Akay et al., the mean age of 445 patients who had traffic crashes was 36.3 years [16]. In our study, the mean patient age was 39.9 years. The likelihood of traffic crashes was higher in the younger age group. Individuals in this age group are in the most active period of their lies and often engage in risky behaviors. This leads to an increase in the possibility of a crash-related injury. Several studies from different regions of the world have reported that traffic crashes are most common in the younger age group [17-19].

In a study by Eliacik et al. of patients presenting to the pediatric ED, 54.3% of the 1,282 injuries analyzed were MVIs. In the same study, 33.1% of the patients were brought from the scene [20]. In our study, 40.5% of the injuries were MVIs and 81.2% of the injured people were brought directly from the crash scene. 

In our study, 64% of the patients were discharged from the ED, while 36% were referred to the other clinics for surgery. The most consulted groups were orthopedics and neurosurgery departments, which aligns with findings by Varlik et al. [21]. This is an expected result because the most commonly injured regions include the head-neck and the extremities.

According to the literature, traffic crashes are well known to increase in the summer months [22]. In our study, the most common season in which traffic crashes occurred was summer by 33.7%, which also aligns with findings reported by Eliacik et al. [20]. We believe that the increased rate of traffic crashes in summer was a result of more common engagement in the outdoor environment and increased travel in this season due to holiday. In a study by Erenler et al. analyzing traffic crashes in Turkey between 2013 and 2017, crashes were most common in August and least common in February [23]. Similarly, in our study, crashes most commonly occurred in August (12.5%) and least commonly in February (3.1%).

Kalafat et al. evaluated the impact of Ramadan on traffic crashes and found the mean number of crashes was slightly higher in Ramadan compared to the non-Ramadan months. In addition, they found that the rate of driving with drinking was lower in Ramadan compared to the other periods [24]. In our study, the rate of traffic crashes significantly decreased during the Ramadan month. In a study by Khammash and Al-Shouha, the rate of traffic crashes was also lower during the Ramadan month [25]. Results from Ramadan month studies may differ from studies using Christian months. For example, the study by Kalafat was conducted in August, in which air temperatures are the highest for the year. In August, fasting people are anxious and stressed due to the heat, and psychomotor performance is decreased [24]. In contrast, our study and the study by Khammash were conducted in a season in which the air temperatures are relatively mild [25].

One of the most important factors affecting traffic crashes is alcohol consumption. In a study from Italy, alcohol was detected in the blood of 56.7% of the persons involved in motor vehicle crashes [26]. In a study by Akay et al., blood alcohol level was >0.5% in 23.4% of the participants who were involved in a traffic accident [16]. In our study, we found that the rate of crashes involving alcohol consumption was 14.5%. The differences between the incidences in alcohol-related traffic crashes may be due to differences in alcohol use habits and frequency of alcohol intake between countries and cultures.

Study limitations

Our study was not without some important limitations. The retrospective design of our study caused difficulties in assessing the clinical data of some patients. In addition, parameters evaluated during the first admission, such as patients’ affected body regions, admission Glasgow Coma Scale scores, and vital findings, could not be included. However, we believe that our results will further contribute to the literature because of the necessity of sustainable analysis of traffic crashes from the viewpoint of EDs.

## Conclusions

Traffic crashes that cause a significant workload in EDs and increase ED crowds are predictable and, thus, preventable events. Close coordination and collaboration between many sectors and disciplines using a holistic and integrated approach is needed to overcome the problem of overburdening EDs with motor vehicle crash-related injuries. In this study, we analyzed several parameters that can affect MVIs and PSs. According to the results of our study, traffic crashes occur more commonly among young men and in the summer season, while national, official, and religious holidays were not related to the frequency of traffic crashes. Emergency medical professionals should have continuous education about basic and advanced trauma, given the proportion of trauma-related presentations. Because the etiology of traffic crashes varies depending on lifestyle and conditions, further similar studies with a large series of patients should be continuously performed, and available data on this issue should be updated regularly.
